# *Epilepsia Open*—February 2026 Announcements

**DOI:** 10.1002/epi4.70228

**Published:** 2026-02-13

## ILAE CONGRESSES

19th Latin American Summer School on Epilepsy

February 23–28, 2026

Guadalajara, Mexico

Curso virtual: Correlación anátomo‐electro‐clínica

March 22–May 2, 2026

Online

8th ILAE School on EEG in the First Year of Life

March 30–April 2, 2026

Cambridge, UK

5th International Summer School of Neuropsychology

April 19–24, 2026

Picardy, France

8th East Mediterranean Epilepsy Congress

April 30 –May 2, 2026

Al Khobar, Saudi Arabia

ILAE School on Neuroimaging 2026

May 6–9, 2026

Potsdam, Germany.

XIV Congreso Latinoamericano de Epilepsia

May 16–18, 2026

Lima, Peru

ILAE Summer School on EEG and Epilepsy

July 11–19, 2026

Dianalund, Denmark & Online

1° Curso Latinoamericano de EEG Pediátrico

August 6–9, 2026

TBC

16th European Epilepsy Congress

September 5–9, 2026

Athens, Greece

16th International Summer School for Neuropathology and Epilepsy Surgery

September 10–13, 2026

Erlangen, Germany

16th Asian & Oceanian Epilepsy Congress

November 19–22, 2026

Kuala Lumpur, Malaysia

## 2027

37th International Epilepsy Congress

August 28–September 1, 2027

Amsterdam, Netherlands

## ILAE WEBINARS

Hemimegalencephaly: From Diagnosis to Surgical Treatment ‐ A Multidisciplinary Approach

February 6, 2026

Optimisation du traitement de l'épilepsie

February 10, 2026

Accés aux Médicaments anti Épileptiques en Afrique

February 17, 2026

Status Epilepticus

February 24, 2026

SEEG‐guided Radiofrequency Ablation

February 27, 2026

5th Rome Debate on Developmental and Epileptic Encephalopathies

February 28, 2026

Updated classification of epileptic seizures

March 26, 2026

## OTHER CONGRESSES

Paediatric Epilepsy Training 1—Virtual

February 10, 2026

Online

4th European Congress of Neurology and Neuropsychiatry

February 23–24 2026

Paris, France

Paediatric Epilepsy Training 2—Bristol

February 26–27, 2026

Bristol, UK

Paediatric Epilepsy Training 3—Bristol

February 26–27, 2026

Bristol, UK

4th International Artificial Intelligence in Epilepsy and Neurological Disorders Conference

March 16–19, 2026

San Juan, Puerto Rico

Neuropsychology in Epilepsy Training (NET) India 2026

March 19–21, 2026

Mumbai, India

EPIPED Course: Treatment Strategies in Pediatric Epilepsies

April 22–25, 2026

Girona, Spain

18th Eilat Conference on New Antiepileptic Drugs and Devices

May 3–6, 2026

Madrid, Spain

International Conference for Post‐Traumatic Epilepsy 2026

May 3–6, 2026

Princeton, New Jersey, USA

Paros Epilepsy 2026

May 11–15, 2026

Paros, Greece

20th Specialist Epilepsy Teaching Weekend

May 16–17, 2026

Birmingham, UK

13th International Residential Course on Drug Resistant Epilepsies

May 17–23. 2026

Tagliacozzo, Italy

64th Annual Meeting of the German Society for Epileptology

June 10–13, 2026

Würzburg, Germany

Paediatric Epilepsy Training 2—Leeds

June 11–12, 2026

Leeds, UK

Paediatric Epilepsy Training 3—Leeds

June 11–12, 2026

Leeds, UK

22nd San Servolo Advanced Epilepsy Course

July 20–31, 2026

San Servolo (Venice), Italy

Epilepsy Imaging in Resource Strained Settings

August 31–September 12, 2026

TBC

1st Swiss Neuro Week

November 18–20, 2026

Bern, Switzerland

